# Electrocardiogram Acquisition Errors or Myocardial Infarct

**DOI:** 10.1155/2011/605874

**Published:** 2011-09-04

**Authors:** Sari U. M. Vanninen, Kjell C. Nikus

**Affiliations:** Department of Cardiology, Heart Center, Tampere University Hospital, PL 2000, 33521 Tampere, Finland

## Abstract

Incorrect lead placement may result in unnecessary therapeutic interventions. We present a case report of 53-year-old man with new inferior T-wave inversions in the 12-lead electrocardiogram (ECG) noted during routine followup of hypertension without any cardiovascular symptoms.

## 1. Introduction

The common right arm/left arm lead switch is easily recognizable, but many other lead placement errors are possible. The less common ones are often difficult to detect, but errors are quite important clinically, as they may induce erroneous diagnoses [1]. The proposed technical modification has the potential to minimize the number of errors in electrode placement, with subsequent savings in money, time, and valuable data [2].

## 2. Case Report

A fifty-tree-year old man with new T-wave inversions in the 12-lead ECG noted during a routine followup for hypertension. The patient also had dyslipidemia, but no history of coronary disease or diabetes. His hypertension was well controlled on a combination of beta-blocker and calcium-blocker; in addition, he used a statin.

**Figure 1:**
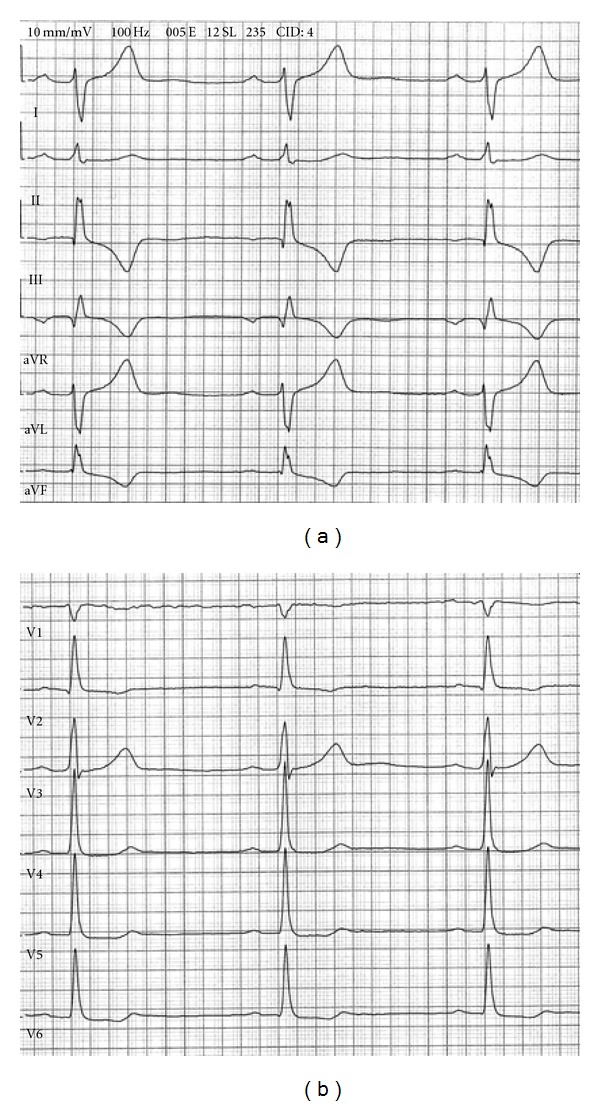
Electrocardiogram with right axis deviation, in leads I and aVL rS complexes with very deep S waves and prominent T waves not usually seen in these leads and more typical of V2 lead. Note the different direction of the ORS complex in lead I and leads V5 and V6.

The physical examination was entirely normal. The ECG obtained sinus rhythm at 54 beats per minute, with rS configuration in lead I and aVL, indicating a left posterior fascicular block and narrow Q waves in III and aVF accompanied by deeply inverted T waves in both leads. In the precordial leads, we observed an abrupt transition in V2 with a qR complex and inverted T wave (Figure 1). Initially, the ECG was interpreted as a recent inferior and a posterior myocardial infarction (MI). 

**Figure 2:**
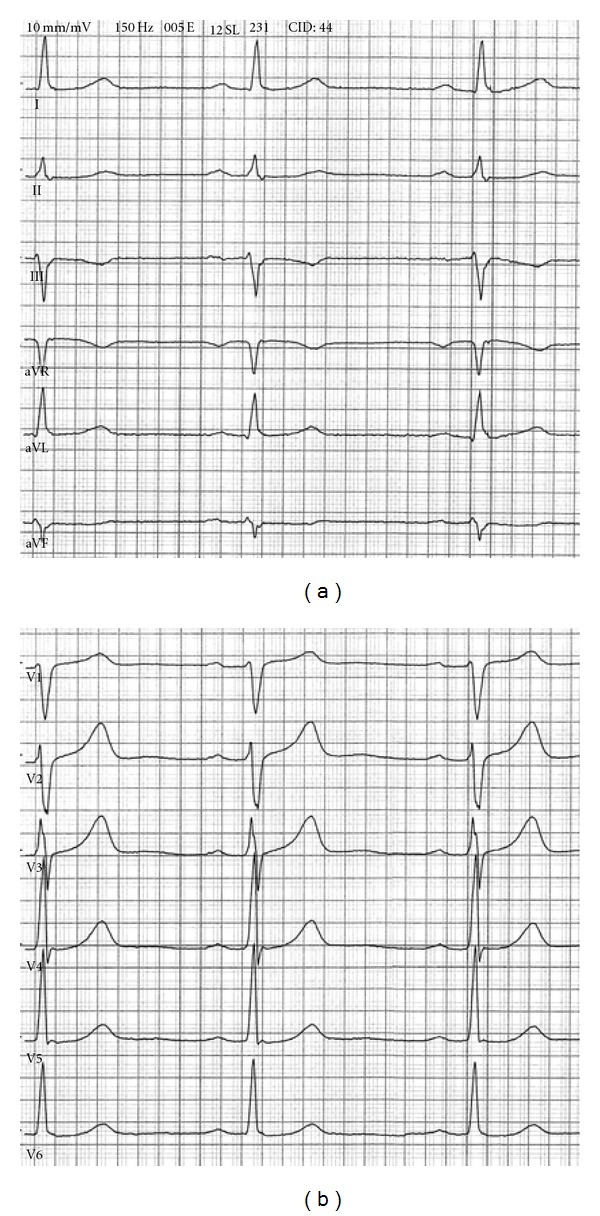
Electrocardiogram from the patient of Figure 1 with electrodes in the right positions. Note that lead II is not modified, given that there was no interchange between electrodes of the right arm and the left leg. The R wave becomes incrementally larger from leads V1 to V6.

Before cardiological checkup, it was decided to perform a new ECG (Figure 2) that proved to be normal with a QRS axis at −13 degrees without deep S wave in aVF and III. Similarly, the transition zone appeared in V3 with a normal R-wave progression and without the qR complex previously seen in V2. This control ECG was similar to an ECG one year earlier. At this point, it was evident that a technical mistake during the ECG acquisition had to be considered.

## 3. Discussion

Interchange between electrodes of the left arm and the left leg in a patient with left anterior fascicular block in the basal ECG may raise the suspicion of inferior myocardial infarction with inverted T waves in leads III and aVF [1, 3]. Cross-over lead interchange between right arm and left leg often produces image of an inferior myocardial infarction with inverted T wave and nonsinus rhythm [1, 4]. 

In our case, there was an abnormal transition in precordial leads with a qR complex morphology in V2 that did not concur with either V1 or V3. If there is a marked discrepancy in the ECG pattern among leads that reflect electrical activity in a similar anatomical location, for example, if lead I and aVL QRS complex is different from that of V5 and V6, lead reversal should be suspected [1].

The ECG morphology registered in the frontal plane in this case can be considered as a mixture of frontal and horizontal planes because of interchange between electrodes of the left arm and V2 [5]. 

Incorrect lead placement may include application of the electrode in a wrong location, connection of the cable to the wrong electrode, or both [3]. The explanation for the accidental interchange between the electrode of the left arm and V2 is that yellow color is present in both electrodes following recommendations of the International Electrotechnical Commission [4, 5].
